# Social cognition and quality of life in Huntington's disease

**DOI:** 10.3389/fpsyt.2022.963457

**Published:** 2022-08-24

**Authors:** Clare M. Eddy, Hugh Rickards

**Affiliations:** ^1^BSMHFT National Centre for Mental Health, Birmingham, United Kingdom; ^2^Institute of Clinical Sciences, College of Medical and Dental Sciences, University of Birmingham, Birmingham, United Kingdom

**Keywords:** apathy, emotion, empathy, Huntington's disease, quality of life, social cognition, social function, Theory of Mind

## Abstract

Individuals with Huntington's disease (HD) and their close others report difficulties with social interaction, and previous studies have shown that the areas of quality of life detrimentally impacted by HD include social and emotional domains. However, despite the finding that people with HD often exhibit difficulties on standard tests of social cognition, the relationship between such impairments and patients' everyday life has remained largely unexplored. We used a range of tasks assessing empathy, emotion recognition and Theory of Mind, to investigate whether patients' performance may predict quality of life within the social and emotional domains, while also accounting for broader cognitive function, behavioural changes, motor symptoms, disease stage and functional capacity. Poorer social functioning was predicted specifically by a reduced tendency to attribute intentionality while viewing social animations, in addition to emotional blunting and apathy, while role limitations due to emotional problems were predicted by personal distress, irritability and aspects of executive function. These findings highlight the potential impact of Theory of Mind impairment on quality of life in HD, and suggest that enhanced assessment of social cognition will offer unique insight into patients' social function and related wellbeing.

## Introduction

The inherited neurodegenerative disorder Huntington's disease (HD) features a range of cognitive and behavioural difficulties, such as executive deficits and changes to mood and temperament, as well as characteristic motor dysfunction. These difficulties can affect the wellbeing of patients and their close others as the disease progresses. Quality of life (QoL) relates to an individual's perception of their position in life, within the context of the culture and value systems in which they are living, and in relation to their personal goals, expectations, standards and concerns (1). Previous studies have shown that health related QoL is generally lowest at later stages of HD and more varied earlier in the disease course (2). Numerous previous studies suggest the most influential factors are likely to be functional capacity and depression (2–4). However, some studies have also suggested that cognition may play a role. For example, Banaszkiewicz et al. (5) found that in addition to depression, cognitive disturbances (assessed *via* the Stroop task, verbal fluency test and Digit Symbol Substitution Test) were the determinants of patients' QoL, while motor disturbances and depression were predictors of caregiver burden.

The areas of QoL that may be impacted by HD include social and emotional domains. This includes the social and emotional aspects of QoL of patients' close others and carers (4), who may be particularly burdened by patients' social withdrawal (6). Helder et al. (7) found that patients with HD exhibited functional impairment according to sickness impact profile (8) scores, as well as severe difficulties in many aspects of QoL including social interaction and emotional behaviour. In this study, only 11% of the psychosocial dimension of QoL was linked to motor symptoms, functional capacity or cognitive function (mini-mental state exam), suggesting the influence of additional variables. A later study (9) recommended using the SF36 (10) as a generic QoL instrument in HD, finding it was less influenced by motor symptoms and had greater construct validity and test-retest reliability. While this latter study found that psychosocial aspects of QoL were particularly affected by HD, communication impairments were also suggested to be important, highlighting the need for thorough evaluation of patient's cognitive impairments in relation to broader QoL scores.

The cognitive impairments experienced by those with HD include difficulties with social cognition. These can take the form of deficits in understanding and explaining social interactions and individuals' mental states (11–14), and recognising others' emotions from facial expressions and body posture (11, 15–19). Sometimes deficits on social cognitive tasks are correlated with executive deficits and/or motor symptoms (12, 17, 20, 21). Having said this, they are unlikely to be completely explained by them, given that studies using control tasks can indicate selective impairment in emotion related reasoning (22). Moreover, social cognitive impairment can arise early in HD before motor signs or severe cognitive decline (18, 23), as well as being correlated with reduced functional capacity (17, 24). Patents' impairments in social cognition align with clinical observation (25) and patient and carer reports (26, 27) highlighting interpersonal difficulties which imply a lack of empathy (26) and involve offensive or antisocial behaviour (28–30). Yet while it is likely that social cognitive impairments contribute to early ‘personality changes' (31) and related interpersonal problems (32), there has been surprisingly little scientific exploration of how difficulties on traditional tests of social cognition may impact QoL or translate into everyday social problems in HD.

In the current study, we explored specific associations between social cognitive performance on a variety of traditional neuropsychological measures assessing empathy, emotion recognition, Theory of Mind (ToM) and QoL within the emotional and social domains. To help disentangle effects of social cognition from the broader impact of clinical status, we aimed to include a wider variety of cognitive measures than previous studies, along with recommended measures to assess behavioural problems, disease burden, motor symptoms, and functional capacity. We hypothesised that impairment in at least some aspects of social cognition would be independently related to the social and emotional domains of QoL, aside of patients' broader cognitive, motor and behavioural symptoms.

## Method

### Procedure

The study received all appropriate ethical approvals and participants gave written informed consent. Participants who had received a positive genetic test for HD were recruited through a specialist outpatient Neuropsychiatry service in Birmingham, UK. Given that HD is progressive and QoL is also liable to fluctuate, all measures were taken by the experimenter at a single time point. First, clinical and demographic information was collected, and participants completed a measure of QoL (SF-36). Tests of social cognition assessed ToM (e.g., mental state attribution), recognition of socially inappropriate behaviour, recognition of mental states from facial expression and emotional reactivity towards others (eight measures). To help disentangle the influence of cognition more generally, we included seven measures of executive function that assessed verbal fluency, task switching, response inhibition and working memory plus one task assessing spatial perspective taking. To explore relationships with broader emotional problems, we included a measure of alexithymia as well as a disease specific interview for the assessment of mood and behavioural problems (six scores).

We also included 3 key clinical measures relating to disease progression in the form of disease burden (calculated by CAG repeat number – 35.5, × age), motor symptoms and total functional capacity (TFC). TFC scores (33) constitute a clinician-rated assessment of independence, occupation and the ability to manage finances, household duties and other activities of daily living (better functional capacity is indicated by higher raw scores and lower stage scores). The Unified Huntington's Disease Rating Scale (UHDRS) (34) motor assessment is used to assess severity of motor symptoms and involves observation of the patient's ability to complete motor tasks such as tandem walking, ocular tracking, and hand movements. Scores can range from 0 to 124 and higher scores indicate more severe motor impairments. It can also be used to give an indication of diagnostic confidence level (DCL).

### Sample

The sample contained 32 participants (17 females, 15 males) with genetically determined HD, aged from 37 to 66 (mean 53.47, SD 7.55), UHDRS total motor scores ranging from 0 to 71 (mean 28.28, SD 17.39), DCL from 1 to 5 (mean 3.31, SD 1.23), disease burden from 154–529 (mean 386.90, SD 97.29) and TFC stage scores from 1 to 3 (mean 2.06, SD 0.80). More specifically: DCL 1 = three patients, with motor scores from 0 to 2; DCL 2 = four patients, with motor scores from 5 to 8; DCL 3 = twelve patients, with motor scores from 15 to 36; DCL 4 = six patients, with motor scores from 22 to 40; DCL 5 = seven patients, with motor scores from 24 to 71. CAGs were available for 24 participants (40 = 1; 41 = 4; 42 = 5; 43 = 6; 44 = 4; 45 = 3; 47 = 1). Approximately half of the participants were taking medications, the most common of which were antidepressants (e.g., citalopram, fluoxetine, sertraline) and a few were taking tetrabenazine, risperidone or carbamazepine.

### Measures

#### RAND health questionnaire SF36

This self-report Qol measure (10) contains eight subscales: physical functioning, bodily pain, social functioning, energy or fatigue, role limitations due to physical health problems, role limitations due to emotional problems, emotional well-being, and general health perceptions. As our interest was in the potential relationship between social cognition and QoL, we focused on the subscales assessing social function (e.g., To what extent has your health interfered with social activities?), emotional wellbeing (e.g., How much of the time during the past 4 weeks have you felt calm and peaceful?) and role limitations due to emotional problems (e.g., Have you accomplished less than you would like due to emotional problems?). We relied on patient self-report and scoring was based on standard RAND recommendations. Higher scores for each subscale indicate better quality of life. Internal consistency and test-retest reliability for the complete scale has been shown to be sufficiently high in HD (35).

#### The reading the mind in the eyes test

This task (36) assesses recognition of complex mental states from images of the human eye region using a standard set of 36 black and white photographs. Each trial contains four mental state words (forced choice options) around the image e.g., apologetic, friendly, uneasy, dispirited. Participants are asked to select the word they think best matches the image. There is no time limit and a glossary of the mental state terms is available for participants. Higher scores indicate better performance according to the scoring system provided by the authors. In this study, we summed the errors made.

#### Faux pas task

This ToM task (37) includes a series of vignettes which were read to participants, accompanied by presentation of the storey text. In half of the storeys, a character makes a socially inappropriate remark (e.g., one character has just bought some new curtains and another character notices the curtains and happens to say they do not like them), while in the other half, they do not (control storeys). Questions related to identification of the inappropriate remark, comprehension, and storey character mental states are included. For this study, we used the total errors in relation to the recognition score (inaccurate identifications of a faux pas or no faux pas).

#### Animations task: ToM video-clips

The Frith-Happe Animations Task (38, 39) assesses responses to viewing cartoons involving two triangles, which are considered to show social interactions involving mental states (e.g., one triangle coaxing the other triangle), goal directed actions (e.g., dancing), and random movement. Participants are simply asked to comment on what is happening while watching the videos. Participant responses were scored according to the degree of intentions attributed to the shapes, and appropriateness of the overall interpretation. We used the scoring criteria given by the developers, and averaged over the four ToM video-clips (two raters, inter-rater agreement 0.76–0.77). High intention responses often correspond with a high appropriateness score, but not always i.e., many mental state attributions can be made leading to a high intention attribution score, but appropriateness depends on fit with the consensus response.

#### Interpersonal reactivity index

This widely used self-report measure of empathy (40) is thought to encompass both cognitive an affective aspects. Participants decide how well each statement describes them. Each statement falls into one of four subscales: Perspective taking (e.g., When I'm upset at someone, I usually try to “put myself in his shoes” for a while), personal distress (e.g., When I see someone who badly needs help in an emergency, I go to pieces), fantasy (e.g., I really get involved with the feelings of the characters in a novel) and empathic concern (e.g., I often have tender, concerned feelings for people less fortunate than me). All four individual subscales were included in the current study.

#### FAS test and controlled oral word association test

For the FAS test participants were asked to say out loud as many words as they can think of beginning with a given letter, apart from proper names. Participants were given 1 min to respond to each of three letters (F, A, and S) in turn. For the Controlled Oral Word Association Test (41) three categories (fruit, animals, and vegetables) were used as the prompts instead of letters. Examples were offered e.g., for the letter C, you could say church, choose, cake etc. Words were summed for each task and higher scores indicate greater verbal and semantic fluency.

#### Digit symbol substitution test

Participants used a coding system (42) in which the numbers from 0 to 9 correspond to simple symbols (e.g., =). They filled in a rows of boxes containing numbers with the matching symbols, from left to right. Scores indicate the number of correct items completed in 2 min and higher scores therefore indicate better performance.

#### Trail making test

For the first part of the task (43), participants drew lines to join small circles that each contained a number (1–25) spread out over a page, in ascending order (from 1–2, 2–3, etc.). For the second part, the circles contained the numbers 1–12 and letters A–L. This time participants joined the circles alternating from number to letter, in ascending order (1-A, A-2, 2-B, etc.). Time to completion was recorded. As scores represent time differences between baseline and the test (switching) condition, higher values represent greater interference and therefore poorer task shifting performance.

#### Stroop test

Participants first completed the baseline condition, naming the ink colours one by one of a page of 40 series of XXXs, going across the rows from left to right. For the second (test) condition, stimuli were colour names written in nonmatching coloured inks (e.g., “green” shown in red ink). Errors and time taken for each condition were recorded (44). Time scores represent time differences between baseline and the test (inhibition) condition, with higher values reflecting greater interference and therefore poorer performance.

#### Digit ordering test-adapted

Participants were asked to recall a mixed string of digits (e.g., 3,7,4,8) in ascending order immediately after being read out by the experimenter (45). There are pairs of strings for each length, sometimes with a repeated digit included. The test ends when two successive trials are answered incorrectly. Scores represent the longest string correctly responded to, with half a point deducted if only one string of that length was answered correctly.

#### Spatial perspective taking

This task was included because it was previously found to be correlated with social cognition in HD (17). During this task, objects were placed in a set position in front of each participant. The corresponding test card featured images of the object from four different views e.g., the participant's view, the experimenter's view etc. Set questions tested the ability to select the correct perspective of each object from different viewpoints.

#### Toronto alexithymia scale

This scale (46) was designed to assess alexithymia i.e., difficulties identifying and expressing one's emotions. Participants used a Likert scale to express how much they agreed or disagreed with statements such as “I find it hard to describe how I feel about people”. We used the total scores from this self-report measure.

#### Problem behaviours assessment (short-form)

This short interview (47) designed for HD is recommended for the assessment of a range of behavioural symptoms that are commonly experienced by patients (48). We used severity scores based on previous recommendations (49) and excluded the subscales linked to disorientation and psychosis as these were not reported by more than 2 patients within our sample. This left severity scores for the subscales depression, anxiety, irritability, aggression, apathy and perseveration.

### Statistical analysis

Correlations were calculated between each of the three QoL subscales and all cognitive, social cognitive and behavioural measures, plus three key clinical scores (DB, UHDRS TMS and TFC). Normality tests indicated skewed distributions for many variables, therefore Spearman's correlations were calculated. Stepwise regressions were then conducted treating the SF36 as a continuous measure (50) using each QoL subscale as DV, including all significantly correlated variables (*p* < 0.05) as IVs. The best models were determined based on the highest proportion of variance explained and all predictors making a statistically significant contribution to the model.

## Results

Quality of life scores for individuals with HD ranged from 25 to 75 (mean = 73.05, SD = 27.52) for social functioning, 28–64 (mean = 63.88, SD = 18.58) for emotional wellbeing and 0–100 (mean = 52.08, SD = 45.55) for role limitations due to emotional problems. For comparison, these scores were clearly below the norms reported for the general population (51) and those reported in the Medical Outcomes Study which included patients with physical health conditions such as hypertension and/or depression (10) (social functioning mean = 78.77, SD = 25.43; emotional wellbeing mean = 70.38, SD = 21.97; role limitations due to emotional problems mean = 65.78, SD = 40.71). In relation to HD, the scores for the current sample were higher for social function but lower for emotional wellbeing and role limitations due to emotional problems when compared to those found in a previous study (3) (social functioning mean = 61.75, SD = 22.13; emotional wellbeing mean = 71.70, SD = 19.58; role limitations due to emotional problems mean = 57.62, SD = 48.13).

Correlations are shown in Table 1. Social functioning QoL was significantly negatively correlated with all three clinical scores (motor symptoms, TFC, disease burden), most scores on executive measures, and scores on social cognitive tasks but not on the Interpersonal Reactivity Index (IRI). Emotional wellbeing QoL was correlated with numerous social cognitive measures and behavioural symptoms, but no key clinical scores or measures of executive function. QoL scores for role limitations due to emotional problems were related to many measures (social cognition, executive function, behaviour), but not clinical measure scores. Social functioning was the only QoL domain related to motor symptoms and functional capacity, emphasising relevance to the global picture of HD.

**Table 1:** Correlations between quality of life domains and all other measures.

**Measure**			**Social functioning**	**Emotional wellbeing**	**Role limitations (emotional problems)**
Clinician rated	Disease burden		−0.291, 0.158	0.062, 0.768	−0.213, 0.306
	Total Functional Capacity score		−0.439, 0.012^*^	−0.081, 0.660	−0.346, 0.053
	Motor symptom severity		−0.486, 0.005^*^	−0.036, 0.844	−0.292, 0.104
Cognition/executive function	FAS verbal fluency test		0.166, 0.007^*^	0.286, 0.113	0.396, 0.025^*^
	Controlled Oral Word Association Test		0.489, 0.005^*^	−0.053, 0.774	0.335, 0.061
	Stroop task	Errors	−0.248, 0.178	−0.003, 0.975	−0.059, 0.751
		Times	−0.236, 0.200	0.119, 0.523	−0.130, 0.487
	Trail Making Test		−0.526, 0.002^*^	−0.199, 0.523	−0.532, 0.002^*^
	Digit Ordering Test-Adapted		0.539, 0.001^*^	0.314, 0.080	0.561, <0.001^*^
	Digit Symbol Substitution Test		0.452, 0.011^*^	0.115, 0.536	0.361, 0.046^*^
	Spatial Perspective Taking task		−0.153, 0.413	−0.037, 0.845	0.031, 0.869
Social cognition	RMET (errors)		−0.493, 0.004^*^	−0.130, 0.479	−0.348, 0.051
	Faux pas recognition (errors)		−0.168, 0.359	−0.176, 0.337	−0.355, 0.046^*^
	Animations (Theory of Mind)	Intentions	0.590, <0.001^*^	0.043, 0.817	0.408, 0.020^*^
		Appropriateness	0.365, 0.040^*^	−0.021, 0.911	0.285, 0.113
	Interpersonal Reactivity Index	Perspective taking	0.212, 0.243	0.352, 0.048^*^	0.287, 0.112
		Personal distress	−0.230, 0.204	−0.486, 0.005^*^	−0.515, 0.003^*^
		Empathic concern	0.277, 0.125	0.265, 0.142	0.058, 0.754
		Fantasy	−0.197, 0.280	−0.455, 0.009^*^	−0.371, 0.037^*^
Emotional/behavioural problems	Toronto Alexithymia Scale		−0.529, 0.002^*^	−0.207, 0.255	−0.205, 0.261
	Problem Behaviours Assessment-short (severity)	Depression	−0.282, 0.118	−0.605, <0.001^*^	−0.478, 0.006^*^
		Anxiety	−0.387, 0.029^*^	−0.539, 0.001^*^	−0.583, <0.001^*^
		Irritability	−0.476, 0.006^*^	−0.400, 0.023^*^	−0.560, <0.001^*^
		Aggression	−0.146, 0.142	−0.484, 0.005^*^	−0.431, 0.014^*^
		Apathy	−0.604, <0.001^*^	−0.239, 0.188	−0.390, 0.027^*^
		Perseveration	−0.374, 0.035^*^	−0.290, 0.107	−0.420, 0.017^*^
Quality of life	Social functioning		X	0.362, 0.042^*^	0.516, 0.003^*^
	Emotional wellbeing		0.362, 0.042^*^	X	0.559, <0.001^*^
	Role limitations (emotional problems)		0.516, 0.003^*^	0.559, <0.001^*^	X

KEY, Spearman's Rho; p-value; RMET, Reading the Mind in the Eyes Test.

* Significant at p < 0.05 therefore included in regression modelling.

Stepwise linear regression analysis revealed that 56% of the variance in the social functioning QoL subscale (*F*(3, 27) = 13.784, *p* < 0.001) could be predicted by the variables apathy (β = −0.441, *p* = 0.002), ToM intention (β = 0.375, *p* = 0.006) and Toronto Alexithymia Scale (TAS) scores (β = 0.273, *p* = 0.047). Better social functioning was predicted by lower apathy, greater intention attribution (see Table 2 for example participant responses related to the ToM intention measure), and lower alexithymia. The best model for the QoL subscale role limitations due to emotional problems predicted 63% of the variance (*F*(3, 27) = 18.063, *p* < 0.001) and included the variables irritability (β = −0.475, *p* < 0.001), IRI personal distress (β = −0.407 *p* = 0.001) and Trail Making Test (TMT) times (β = −0.365, *p* = 0.004), showing that greater personal distress, behavioural difficulties, and poorer cognition negatively impacted this aspect of role fulfilment. The best model for the QoL subscale emotional wellbeing predicted 43% of the variance (*F*(2, 29) = 12.823, *p* < 0.001) and included the variables depression severity (β = 0.369, *p* = 0.026) and the QoL subscale role limitations due to emotional problems (β = 0.418, *p* = 0.013).

**Table 2:** Intention attribution in response to Animations Task Theory of Mind video-clips.

**Animation theme**	**High intention response**	**Low intention response**
Coaxing	“*trying to encourage to come out, big is mother and little baby… showing, teasing the little one out”*	“*circling, came out of box, circling, red one in box”*
Seduction	“*big one won't let him out, trying to find a way out… tricked him and escaped”*	“*red and blue triangles, blue touching red, dancing around, he's gone off the page”*
Teasing	“*blue is following red, but doesn't want red to know he's following him, he's spying on him”*	“*red comes in, blue behind, going same direction, up and across, turning round”*
Surprising	“*like a game of hide and seek, they couldn't see each other, the red is inside and the blue knocked on the door and hid”*	“*box, closed, with large one, opened door to get out and small one got in”*

To offer additional insight into the relationship between social functioning and apathy, we checked whether apathy severity was also directly correlated with social cognition, alexithymia (given a potential emotion processing overlap), and TMT scores (given the potential associations between cognitive shifting, apathy and perspective taking). Apathy severity was correlated with the Reading the Mind in the Eyes Test (RMET; *r* = 0.509, *p* = 0.002), TAS (*r* = 0.309, *p* = 0.028) and TMT (*r* = 0.400, *p* = 0.023) scores.

## Discussion

We found that social functioning was predicted by the tendency to attribute intentions while watching ToM animations (see Table 2), suggesting that specific aspects of social cognition may make a unique contribution to QoL in HD. Not all responses with high intention ratings were highly appropriate, and response appropriateness was not a significant individual predictor. One explanation for why this select ToM measure was predictive of social function relates to motivation, given that the task measured spontaneous attribution of mental states in response to the movements of two triangles [see (38, 39)]. Motivation to attend to social cues could influence everyday social functioning in addition to lab task performance. This interpretation would be in line with the predictive value of apathy in relation to social functioning scores, a finding that is in accordance with previous studies linking emotion recognition and social roles to apathy (52, 53). Up to 90% of those with HD experience apathy (54, 55) and it can occur early in the disease course (56), so it will be important to bear in mind motivational factors in future studies of social cognition in HD. Apathy has been linked to damage within subcortical and cortical regions in HD, including emotional-related prefrontal, temporal, and limbic areas such as the amygdala (57), which may suggest dysfunction within a common emotional component, an interpretation in line with the correlation we found between apathy and alexithymia. Furthermore, we found a correlation between apathy and the RMET, while a previous study, highlighted an association between apathy and recognition of happy facial expressions (58). Alternatively, relationships between apathy and social skills could reflect a more cognitive overlap, given that difficulty considering multiple perspectives and possibilities may help to explain help to explain both apathy and impaired social cognition, supported by the correlation between apathy and TMT shifting scores.

Similarly, the predictive value of alexithymia may lie in motivation towards understanding and reflecting upon internally experienced mental states. For example, one recent study found that aspects of emotional insight were associated with apathy in HD (59), although IRI scores were not correlated with social functioning. This could be because this self-report measure asks about general tendencies towards empathy, whereas the other social cognitive tasks gave an indication of current, more objective social cognitive performance. Alternatively, given that RMET performance was linked to social function but faux pas recognition was not, perhaps visual tasks are more closely related to everyday social function in HD, because verbal tasks are more easily influenced by general cognition e.g., working memory demands.

Some of our findings highlight how the contribution of social cognition can be difficult to disentangle from broader cognitive abilities when investigating QoL. While numerous social cognitive measures were correlated with the QoL subscale limited role functioning due to emotional problems, it was only personal distress, plus scores for set-shifting, semantic fluency, and irritability, that predicted how QoL in different contexts was affected by emotional state. Possibly, the common thread was cognitive control, which could affect the experience of emotional distress as well as being relevant to tasks involving attention shifting. Irritability could similarly involve difficulties with shifting attention away from a negative experience, but this should be explored further. It is interesting that irritability may be more important than apathy in predicting role functioning, but one possible explanation is that the influence of carer support may help to reduce the effect of apathy in some contexts.

The emotional wellbeing QoL subscale was not correlated with executive function, but was related to a few measures of social cognition, as well as behavioural symptoms (depression, anxiety, irritability, aggression), which may be expected. However, it was only predicted by depression and the QoL subscale limited role functioning due to emotional problems. The current study therefore suggests that individual behavioural features related to mood and temperament my differentially influence QoL. In summary, a specific relationship between social function and apathy could lie in the ability to shift perspectives, while emotion control may most affect role function and emotional wellbeing.

We chose to use a continuum approach across our sample given the heterogeneous nature of HD, and also because while it may be considered possible to make a meaningful differentiation based on the presence of motor symptoms, these do not necessarily predict other aspects of HD symptomatology, such as cognitive status, which is pertinent to our study. Follow-up research on a larger sample is needed to offer insight in relation to any effects dependent on disease stage, and to allow for additional analyses such as structural equation modelling, in order to better understand the relationships between variables. Other limitations of this study include the range of measures used. For example, we used a generic measure of QoL, rather than the disease specific measure available for HD (60). There can also be issues with self-report measures, which could be influenced by loss of insight, a difficulty which is thought to be particularly relevant to HD (61). Therefore collection of data through additional measures (e.g., carer report, daily activities, relationship satisfaction etc.) would enhance future investigations into the relevance of social cognition to patient and close-other's everyday lives.

In conclusion, aspects of ToM may make a significant independent contribution to social functioning in HD, aside of other cognitive and behavioural difficulties. While more research is needed, our findings imply the likelihood of some early changes to patients' social skills, and the potential for these to impact QoL. They further highlight the importance of assessing motivation in addition to ability when investigating the impact of social cognition on patient and carer wellbeing, and compel the development of more relevant and ecologically valid measures for the assessment of everyday social skills in HD.

## Data availability statement

The raw data supporting the conclusions of this article will be made available by the authors, without undue reservation.

## Ethics statement

The studies involving human participants were reviewed and approved by South Birmingham REC. The patients/participants provided their written informed consent to participate in this study.

## Author contributions

CE conceived and designed the study, collected the data, analysed the data, and wrote the first draft of the manuscript. HR supported data collection, and reviewed and edited the manuscript. Both authors contributed to manuscript revision, read, and approved the submitted version.

## Funding

The data used in this study forms part of a larger project funded by the European Huntington's Disease Network Project 297.

## Conflict of interest

The authors declare that the research was conducted in the absence of any commercial or financial relationships that could be construed as a potential conflict of interest.

## Publisher's note

All claims expressed in this article are solely those of the authors and do not necessarily represent those of their affiliated organizations, or those of the publisher, the editors and the reviewers. Any product that may be evaluated in this article, or claim that may be made by its manufacturer, is not guaranteed or endorsed by the publisher.
